# Chromosomal Instability in Chronic Myeloid Leukemia: Mechanistic Insights and Effects

**DOI:** 10.3390/cancers14102533

**Published:** 2022-05-21

**Authors:** Jayastu Senapati, Koji Sasaki

**Affiliations:** Department of Leukemia, The University of Texas MD Anderson Cancer Center, Houston, TX 77030, USA; jsenapati@mdanderson.org

**Keywords:** CML, chromosomal instability, additional cytogenetic abnormalities

## Abstract

**Simple Summary:**

Chronic myeloid leukemia is a disease diagnosed by the presence of the Philadelphia chromosome, which leads to the BCR::ABL fusion oncoprotein and overactive tyrosine kinase activity. Multiple other genetic aberrations and chromosomal changes make the disease very heterogeneous, and these changes increase as the disease becomes more aggressive. Understanding the cause and effects of chromosomal instability in CML might help to improve treatment options and monitoring of patients with advanced phases of CML.

**Abstract:**

The most recent two decades have seen tremendous progress in the understanding and treatment of chronic myeloid leukemia, a disease defined by the characteristic Philadelphia chromosome and the ensuing BCR::ABL fusion protein. However, the biology of the disease extends beyond the Philadelphia chromosome into a nebulous arena of chromosomal and genetic instability, which makes it a genetically heterogeneous disease. The BCR::ABL oncoprotein creates a fertile backdrop for oxidative damage to the DNA, along with impairment of genetic surveillance and the favoring of imprecise error-prone DNA repair pathways. These factors lead to growing chromosomal instability, manifested as additional chromosomal abnormalities along with other genetic aberrations. This worsens with disease progression to accelerated and blast phase, and modulates responses to tyrosine kinase inhibitors. Treatment options that target the genetic aberrations that mitigate chromosome instability might be a potential area for research in patients with advanced phase CML.

## 1. Introduction

Chronic myeloid leukemia (CML) is driven by the juxtaposition of the Abelson leukemia kinase (ABL) proto-oncogene on chromosome 9 onto the breakpoint cluster region (BCR) gene on chromosome 22 by balanced translocation, creating the signature Philadelphia chromosome (altered chromosome 22) and the BCR::ABL fusion gene [1,2]. Driven by constitutively active tyrosine kinase secondary to the fusion gene product, CML is characterized by the accumulation of immature cells in the bone marrow, blood, and spleen, due to the proliferative advantage from the oncogene addiction and differential block [3]. However, CML has been found to have a more heterogeneous genetic signature than previously thought, which involves changes other than the BCR::ABL fusion [4]. Though the Philadelphia chromosome and the ensuing oncogenic overdrive dictates the pathophysiology of CML, there is progressive genetic instability in patients who have transformation of CML to the advanced phases: accelerated phase (AP) and blast phase (BP) [5]. Important considerations in that regard are the additional chromosomal abnormalities and genetic instability, mediated by the heightened sensitivity to genetic stressors and inadequate DNA repair [6,7].

The additive effect of ongoing chromosomal instability, mediated by the upregulated BCR::ABL kinase pathway, leads to further mutations in the genome and epigenome which can ultimately lead to disease transformation into the advanced phases [8,9,10,11]. Though the use of tyrosine kinase inhibitors (TKI) significantly reduces the extent of genetic instability and mutational stress in CML, escape mechanisms and BCR::ABL oncogene-independent genetic aberrations in the relatively quiescent leukemic stem cell population can render these therapies ineffective in a subset of patients [12]. In this review, we will attempt a focused discussion regarding chromosomal instability in CML at baseline and during progression to the advanced phases, examining how it affects leukemogenesis, and its importance in therapy considerations. Though CML was one of the earliest cancers in which the definite pathogenic pathway was delineated, research has exceedingly shown that leukemogenesis in CML can be more complicated than previously thought. Significant progress has already been achieved in the treatment of CML, where new insights are helping to improve therapy development. For example, superior combinations are being formulated in patients with advanced phase CML, while improving responses in patients with chronic phase CML (CML-CP), enabling more patients to achieve treatment free remission (TFR) [13,14,15,16,17,18,19,20].

## 2. Chromosomal Instability in CML

### 2.1. Beyond the Philadelphia Chromosome: Additional Chromosomal Abnormalities

The discovery of the Philadelphia Chromosome [t(9;22)] was a landmark event in the field of oncology, defining a genetic signature that was characteristic of an oncologic process [21]. However, with the evolution of biomedical technology, as we were able to decipher more precisely, it became evident that chromosomal changes in CML can be beyond the Philadelphia chromosome. These aberrations are termed as additional chromosomal abnormalities (ACA) and are now incorporated into both diagnostic and prognostic algorithms for CML. The presence of ACAs at baseline in CML have been associated with poor response rates to TKIs and a higher risk of progression to CML-AP and CML-BP [22,23,24,25,26,27,28,29,30,31,32]. It is important to understand whether these ACAs are the consequence of the exaggerated underlying genetic instability, depicting the aggressive nature of the disease in some patients, or the cause of their inadequate responses. Studies have categorically shown that cells with BCR::ABL fusion have increased propensity for DNA single- and double-strand breaks in response to chemotherapy, reactive oxygen species, radiation, and other triggers [33,34,35,36]. The BCR::ABL oncogene product mediated error-prone DNA repair mechanisms (involving the DNA single-strand adduct and double-strand break repair systems, mismatch repair, and nucleotide excision repair pathways) lead to further chromosomal changes. These include unbalanced re-arrangements, deletions/gains, and copy number variations, which further promote genetic instability [37].

The volume of these ACAs is more common in patients with TKI resistance, being both a cause and effect of disease resistance. Although patients with baseline ACAs might have a poor response to TKIs, the evolution of ACAs can be considered a warning of imminent TKI resistance [25,38,39]. The evolution of ACAs while on TKIs is, thus, considered as a diagnostic criterion for CML-AP by both the European Leukemia Network (ELN) and the World Health Organization (WHO) [40,41]. The latest ELN guideline adds weightage to the presence of high-risk ACAs at baseline, and deems it to be a “warning” with a need for close observation and a change in TKIs if required [40].

Leukemic stem cells, which are usually TKI resistant, provide a fertile ground for progressive genetic instability and are supremely important in disease progression [36,42,43]. The ongoing genetic instability adds to the burden of deleterious mutations in multiple genes that fuel leukemogenesis and transformation of CML into advanced phases. The ACAs are categorized into major or minor route abnormalities based on the frequency of these abnormalities in CML. ACAs which are present in >10% of patients are known as major route, and includes additional Ph chromosome i(17)(q10), trisomy 8, and trisomy 19, while other less frequent ACAs are termed as minor [44,45]. However, this categorization is not precisely prognostic [46]. Certain ACAs, such as 3q26.2 rearrangement, -7/del7q, i(17)(q10), and 11q23 aberrations have an increased risk of disease transformation, as do the total number of ACAs present and the presence of complex karyotypes [24,25]. Irrespective of the generation of TKI used, ACAs have a negative effect on survival in CML [38,44,47]. An analysis of the dynamics of blastic transformation was undertaken at MD Anderson Cancer Center, which studied 2326 patients being treated with TKIs based on their respective ACAs. The frequency of transformation to BP was over 80% in patients with 3q26.2 rearrangements and -7/del 7q, and around 70% in patients with i(17)(q10) or complex karyotypes [48]. The frequency of blastic transformation was 21–34% in patients with other ACAs and 10% in those without ACAs; importantly, patients who had -7/del 7q had the shortest time interval to BP of just 8.1 months, closely followed by 11.9 months in those with 3q26.2 rearrangements and 15.6 months in those with high- risk complex karyotypes. The time from the development of ACAs to blast phase was also dependent on the type of ACA and therapy; this, in turn, had a bearing on overall survival. The above study also shows the important discriminatory power of ACAs in determining the risks of progression to BP, and the need to intervene appropriately.

**Figure 1:**
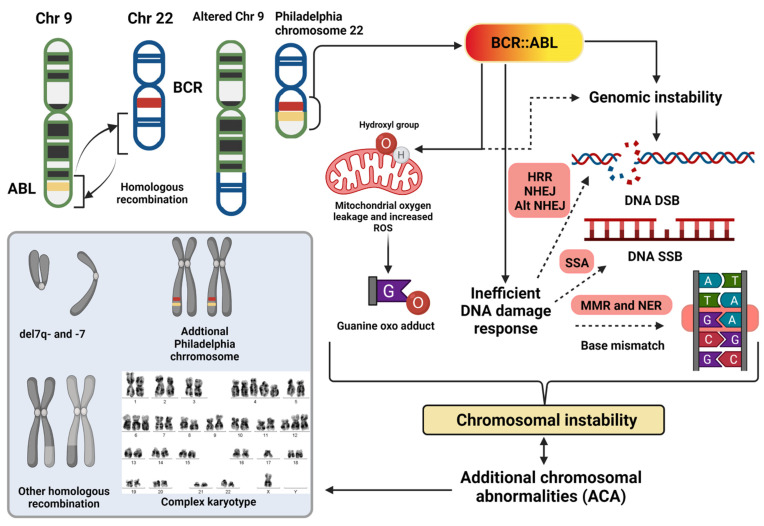
Mechanisms and effect of chromosomal instability in CML. The BCR::ABL fusion oncoprotein leads to a state of genomic instability, along with multiple metabolic changes in the cells, leading to increased production of reactive oxygen species (ROS). There is an increased sensitivity to ROS in the cellular microenvironment in view of defective assessment of genetic stress and aberrations, and a skewed DNA damage response towards error-prone repair pathways such as NHEJ and alternate NHEJ. These ultimately lead to multiple chromosomal changes, such as point mutations in the DNA affecting the genetic and epigenetic pathways alike. Additional chromosomal abnormalities, not limited to the changes in the above figure, are a fallout of these pathological processes which further destabilize the genome. These intertwined steps promote leukemogenesis through proliferation advantage, inhibition of apoptosis, and differentiation block, which results in CML developing more TKI resistance and progression to the advanced phases. G—guanine; A—adenine; T—thymine; C—cytosine; O—oxygen radical; HRR—homologous recombination repair; NHEJ—non homologous end joining; alt NHEJ—alternate pathway of NHEJ; SSA—single-strand annealing; MMR—mismatch repair; NER—nucleotide excision repair; DNA DSB—DNA double-strand breaks; and SSB—single-strand breaks.

As the disease progresses to the advanced phases, the burden of ACAs also increases, highlighting the increasing chromosomal instability and mutational stress. Less than 5% of patients in CML-CP possess ACAs; these numbers increase to around 20–30% in patients with CML-AP, and over 70% in patients with CML-BP [49]. As would be expected, even after the development of BP, ACAs are associated with survival outcomes. Patients without ACAs during their BP have better survival rates compared to those who have ACAs at BP diagnosis, or those who develop them during their BP [48,50]. Thus, the effect of ACAs is additive at each step in the CML disease process; the propensity of these ACAs increases with disease progression, underlining that growing chromosomal instability is important in the pathogenesis of the advanced phases of the disease, and is also a possible effect of the underlying mechanisms which fuel this progression and TKI resistance (Figure 1).

### 2.2. Causes of Chromosomal Instability

We discussed the wide effect of chromosomal instability manifesting as ACAs in CML treatment resistance progression. Chromosomal instability in CML stems from the underlying overactive tyrosine kinase pathway as has been mentioned in the previous section. Multiple mechanisms are at interplay in perpetuating this pathology and we summarize a few of them below.

#### 2.2.1. Reactive Oxygen Species

CML cells have a higher production rate of reactive oxygen species (ROS), due to altered mitochondrial membrane potential and transfer of electrons along the mitochondrial respiratory chain complex [34,51]. The activated phosphatidyl inositol (PI3K)/AKT pathways have been implicated in the excessive oxidative state from mitochondrial dysfunction in CML progenitor cells and have been shown to play a role in the development of TKI resistance [52,53]. The elevated production of ROS in CML cells is coupled with an increased sensitivity of ROS to the DNA in the dividing cells producing multiple genetic aberrations [54]. The ROS (superoxide and hydroxyl group), when not adequately scavenged, causes DNA damage by producing oxo derivatives (of the DNA bases) and DNA double-strand breaks [54,55,56]. Progression of the genomic instability causes further alterations in these oxidative pathways, feeding the system with a more mutagenic milieu [57].

#### 2.2.2. Inefficient Recognition of Genotoxic Stress

CML cells fail to adequately recognize DNA damage from any genotoxic stress due to altered function of the ataxia-telangiectasia and rad 3-related protein (ATR) pathway [58]. The BCR::ABL protein can translocate into the nucleus and, by associating with ATR, it can affect its function and meddle with the mitotic checkpoint by mimicking the action of physiological nuclear c-Abl kinase [58,59,60]. The downregulation of BRCA1 by BCR::ABL is considered to be another mechanism in the deregulation of mitotic checkpoint in response to DNA damage [61]. There remains controversy about whether the BCR::ABL related ATR-Chk1 and ATM-p53-Chk2 axis, which is involved in genomic surveillance, is maintained or perturbed in CML; however, a large number of patients who progress to BP by virtue of wild type p53 downregulation might have this genomic surveillance indeed altered [62]. In patients with BP, increased Bcl-xL and Bcl-2 levels prevent apoptosis and, together with reduced p53 activity, overcome the ATM-p53-Chk2 mediated genomic surveillance and promote leukemogenesis [63].

#### 2.2.3. Error-Prone DNA Repair System

Although elevated ROS is one mechanism of BCR::ABL and alternative kinase pathways mediated DNA damage, the DNA damage repair (DDR) system itself is error-prone in CML, which increases with CML progression. The DDR is set into motion to counteract any DNA damage that occurs during cell division due to exogenous and endogenous stressors, such as ROS, chemotherapy, radiation, or other toxic stimuli [64]. These responses are intricate for the control of progressive genetic instability and disease transformation. In an ideal state, they provide time for corrective repair of the damaged DNA or promote apoptosis of an irreversibly damaged cell.

In CML cells there is a BCR::ABL kinase-mediated error-prone DNA single-strand/double-strand break repair [6]. The mechanisms of DNA strand break repairs in CML include [6,64,65]:

Single-strand annealing [66];

Homologous recombination repair (HRR): slow and error free; occurs during S and G2 phase; and involves BRCA1/2, RAD51 genes;

Non-homologous end joining (NHEJ): rapid and error-prone; works in G1 and early G2 phases; involves Ku70/Ku80, DNA dependent protein kinase, Artemis, and DNA ligase IV (complexed with XRCC4 and XLF); and repair can result in few nucleotide losses at the repair site [67];

Alternative NHEJ: highly error-prone; involves poly ADP ribose polymerase I (PARP I) and DNA ligase IIIα; and repair in the absence of a DNA template leads to large section nucleotide losses/changes and chromosomal translocations emanating from unrelated DNA end ligations by DNA ligase IIIα [68,69,70].

In CML, there are specific aberrations in DSB repair pathways, especially as the disease assumes the blastic phase [71]. With time, there is more dependence on the NHEJ pathways (especially the alternate NHEJ pathway, due to upregulation of DNA ligase IIIα and Werner syndrome related (WRN) protein which facilitate this pathway [68]) compared to the relatively error free HRR pathway, possibly mediated through an upregulation of c-MYC expression by BCR::ABL [7,35,72,73].

When exposed to ionizing radiation, CML progenitor cells have been shown to form unstable chromosomal lesions mediated by breakage–fusion–bridge cycles, which is caused by attempted DNA repair by the NHEJ repair pathway [74]. These unstable lesions were shown to persist even after multiple cellular divisions following the initial radiation exposure in CML progenitor cells compared to normal CD34^+^ cells, highlighting the persistent ineffective DNA repair system in the former. Breakage–fusion–bridge (B-F-B) cycles have been noted to create different types of chromosomal aberrations, including chromosomal amplifications, re-arrangements, copy number variation, and point mutations. B-F-B occurs as the sister chromatids that have lost telomeres in CML cells fuse together on their ends, which are then torn apart during cell division and the process is repeated in the new cell [75,76]. This can also occur when dicentric chromosomes, which are generated by erroneous DSB pathways during DNA repair, are broken during cellular division. The error-prone DDR pathways, in an attempt to repair these broken chromosomes, create further chromosomal changes and instability in CML cells [74].

**Figure 2:**
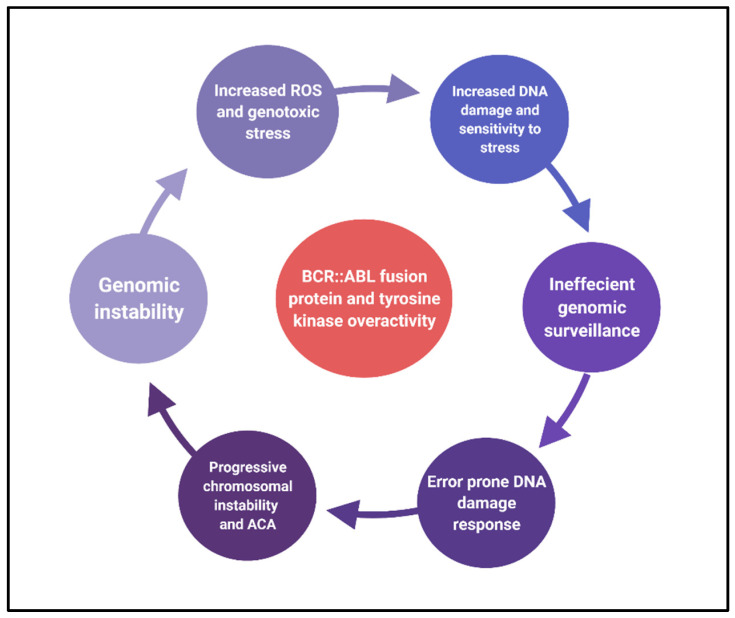
The vicious cycle of genomic and chromosomal instability in CML.

Preclinical studies have shown sensitivity to inhibition of PARP1 and DNA ligase IIIα inhibition in CML cells, given their role in the alternate NHEJ pathway [6,77,78]. Other than DSBs in CML cells, the BCR::ABL overactivity also leads to mismatch repair defects and nucleotide excision repair defects [7,35,37]. Thus, along with the increased production of ROS in CML cells which cause DNA damage, there is a serious defect in DNA repair systems with a shift towards more error-prone repair pathways that enable cytogenetic aberrations and genetic instability. These vicious cycles of DNA oversensitivity to genotoxic stress, poor genetic surveillance, and faulty DNA repair lead to TKI resistance, and promote disease transformation as these processes become widespread through disease progression to AP and BP (Figure 2). Though the aim of the repair mechanisms is to prevent oncogenic stimulus from leading to mutagenic DNA changes, the underlying genomic defects (fueled by BCR: ABL fusion) skew the mechanism to a more nefarious state. This also explains why the burden of the ACAs increases with disease progression in CML.

#### 2.2.4. Centrosomal Aberration

The centrosome is an important part of the microtubule organizing center, and through the production of the mitotic spindles it enables the division of sister chromatids during cell division [79]. Aberrations in the centrosome have been considered a common occurrence in carcinogenesis. Though centrosome aberration leads to chromosomal instability, this might not be enough for the development of cancer [80]. Centrosome aberrations have been found to increase with more advanced disease phases of CML and corelates with the burden of ACAs [81]. Some of the centrosomal aberrations can also be observed in non-hematological tissues in patients of CML being treated with TKIs [82,83]. Centrosomal aberration plays a role in mitigating chromosomal instability in CML and probably works in consort with other pathways, adding to genetic instability. Other mechanisms, such as *Separase* overexpression and hyperactivity, which lead to defective formation of mitotic spindles during cell division, have been described in CML cells as a cause of centromeric dysfunction and aneuploidy and correlate with an increased risk of disease progression [84,85].

### 2.3. Effects of Chromosomal Instability 

Genetic instability in CML is inherent from the leukemic stem cell population stage which is usually resistant to TKI therapy. We discussed in previous sections the factors that lead to chromosomal instability in CML as the disease progresses and are often responsible for therapy resistance. Most of these processes are heavily intertwined. The downstream effect of genetic instability in CML leads to the development of ACAs and aberration in both genetic and epigenetic pathways through gene mutations, alteration in tumor suppressor mechanisms, activation of BCR::ABL independent proliferative pathways, and alterations in drug metabolism [37,86,87,88,89]. Several other kinase pathways, such as the PI3K/AKT pathway, the JAK STAT pathway, and MEK/ERK kinase pathways are activated by the BCR::ABL oncoprotein in CML, and they play a role in drug resistance, disease progression, and maintenance of the leukemic drive [90,91,92]. These mechanisms have been reviewed elsewhere and readers are encouraged to refer to the above references. Importantly, TKIs, by way of suppressing the BCR::ABL signals, are able to reduce mutational and genotoxic stress [5]. However, TKI resistance through mutations in the TKI binding site of the ABL kinase domain, or through other mechanisms, renders the blockade ineffective and sets into motion factors which worsen genetic instability.

Subtle chromosomal aberrations can be deciphered in the form of copy number variations during the process of disease evolution, which parallels the above-mentioned pathologies [93,94]. Other than conventional G-band karyotyping, use of array comparative genomic hybridization or fluorescent in situ hybridization for specific chromosomal changes can be used as more sensitive tools for assessment of chromosomal changes in CML [95,96]. The most direct effects of chromosomal instability can be seen from the impact of ACAs on TKI response, risks of progression to AP/BP, and survival. Though crude, they exemplify the nature of the DNA damage and the faulty DNA repair system that is perpetuated by the oncogenic fusion. Therapeutically, the most important step would be to prevent the onset of ACAs, as ACA evolution on TKIs is worse compared to ACAs at diagnosis of CML [25]. In the presence of high-risk ACAs (described before), the time until blastic transformation can be very short, and urgent alteration of therapy should be instituted to prevent progression.

## 3. Conclusions

There has been tremendous progress in the understanding of the disease biology and treatment of CML over the last two decades. However, with growing knowledge of the disease, it has become apparent that it is not a homogeneous disease process that can be explained by BCR::ABL fusion and overactive tyrosine kinase activity alone. Though the disease-defining event is uniform, there is a heterogenous genetic instability at the heart of the disease which defines the variable therapeutic responses, risks of progression to the advanced phases of the disease, and survival outcomes, even when treated with TKIs. As the disease progresses to AP and BP, it becomes less dependent on BCR::ABL activity. Chromosomal instability is a visible facet of the genetic stress in CML and has important therapeutic and prognostic considerations. Including these ACAs in new and improved treatment models of CML might help us improve therapy options and monitoring to prevent disease progression. Preclinical and clinical studies have shown sensitivity of PARP inhibitors and venetoclax in resistant CML cell lines by inhibiting the error-prone alternate NHEJ pathway; further clinical development is under way for CML [97]. Adequate TKI therapy reduces the risk of cytogenetic evolution and disease transformation, and remains the cornerstone to combat the chromosomal instability in CML. Though patients maintain remission once achieved, the combination of novel agents under clinical development, supportive therapies, and strategies to minimize the risk of toxicities is essential to achieve higher rates of successful TFR [98,99,100,101,102,103,104,105,106].
